# Heterogeneous precipitation mediated heterogeneous nanostructure enhances strength-ductility synergy in severely cryo-rolled and annealed CoCrFeNi_2.1_Nb_0.2_ high entropy alloy

**DOI:** 10.1038/s41598-020-63038-z

**Published:** 2020-04-08

**Authors:** U. Sunkari, S. R. Reddy, B. D. S. Rathod, S. S. Satheesh Kumar, R. Saha, S. Chatterjee, P. P. Bhattacharjee

**Affiliations:** 1 Indian Institute of Technology Hyderabad, Kandi, Sangareddy, 502285 Telangana India; 2 Defence Metallurgical Research Laboratory, Hyderabad, 500058 Telangana India; 3 R&D Division, TATA Steel, Jamshedpur, 831001 India

**Keywords:** Mechanical properties, Metals and alloys

## Abstract

Possibilities of enhancing mechanical properties of brittle intermetallic containing high entropy alloys (HEAs) using novel processing and microstructural design strategies were investigated in the present work. For this purpose, homogenized CoCrFeNi_2.1_Nb_0.2_ HEA consisting of FCC matrix and complex Laves phase particles was successfully processed by severe cold- or cryo-rolling to 90% reduction in thickness followed by annealing (800 °C/1 hour(h)). As compared to cold-rolling, cryo-rolling resulted in a finer lamellar nanostructure and decidedly greater fragmentation of the Laves phase. Upon annealing, the cold-rolled HEA showed a recrystallized FCC matrix dispersed with D0_19_ structured ε nano-precipitates. In contrast, the finer nanostructure and greater driving force for accelerated precipitation of profuse nano-precipitates at the early stages of annealing inhibited recrystallization in the cryo-rolled HEA and resulted in the formation of heterogeneous microstructure consisting of retained deformed and recrystallized regions. The novel heterogeneous microstructure of the cryo-rolled and annealed HEA resulted in a remarkable enhancement in strength-ductility synergy. The present results indicated that cryo-rolling could be used as an innovative processing strategy for tailoring heterogeneous microstructure and achieving novel mechanical properties.

## Introduction

The increase in strength of materials is often accompanied by a concomitant decrease in tensile ductility. This strength-ductility trade-off remains a major challenge for engineering applications of ultrahigh strength materials, which can otherwise offer significant advantages in the form of fabricating lightweight structural components with thinner cross-sections with the additional benefits of materials and energy savings. Therefore, enhancing the strength of materials without compromising ductility, i.e., overcoming the bottleneck of strength-ductility trade-off remains a major focus in materials research. Towards this end, novel alloy compositions which can promote transformation-induced plasticity (TRIP) or twinning-induced plasticity (TWIP) effect have been intensively investigated. More recently, heterogeneous microstructural design, which is featured by intelligently tailoring constituents and domains with wide differences in length scales, hardness or mechanical properties such as bimodal structure^1–3^, harmonic structure^4^, various gradient microstructures including nano-grained^5–9^, nano-domain^10^ and nano-twinned grains^11^, laminate architecture^12,13^, heterogeneous lamellar microstructure^14,15^ and dynamically reinforced heterogeneous structure^16^, has emerged as a potent strategy for synergistic improvement in strength-ductility balance in materials.

Developing heterogeneous microstructures through bulk processing such as thermo-mechanical processing (TMP) treatments comprising of deformation and annealing appear particularly attractive for developing advanced alloys such as high entropy alloys (HEAs)^17^, which are an emerging class of multicomponent alloy systems occupying the central regions of the massive compositional space in the hyper-dimensional phase diagrams with intriguing microstructures and properties for advanced applications^18–22^. Recent investigations have concluded that HEAs processed by innovative TMP treatments can develop novel structurally and compositionally heterogeneous microstructures with outstanding strength-ductility synergy for advanced structural applications^15,16,23–27^.

Nevertheless, achieving desired properties in HEAs containing intermetallic phases remains more challenging^28–35^, but could significantly widen the scope and opportunities for potential structural applications of HEAs. A series of CoCrFeNi_2.1_Nb_x_ HEAs containing Laves phases recently investigated by us show improved mechanical properties after aging treatments due to the fine-scale precipitation of intermetallic D0_19_ structured ε phase^36^. These encouraging results indicate scope for further enhancement in properties in these HEAs using heterogeneous microstructural design through innovative processing strategies but have not been clarified yet. In the present work, we have investigated the effect of severe cold- and cryo-rolling on microstructure and properties of intermetallic containing CoCrFeNi_2.1_Nb_0.2_ HEA. In particular, we have observed the remarkable effect of the cryo-rolling on mechanical properties, which is highlighted in the present research.

**Figure 1:**
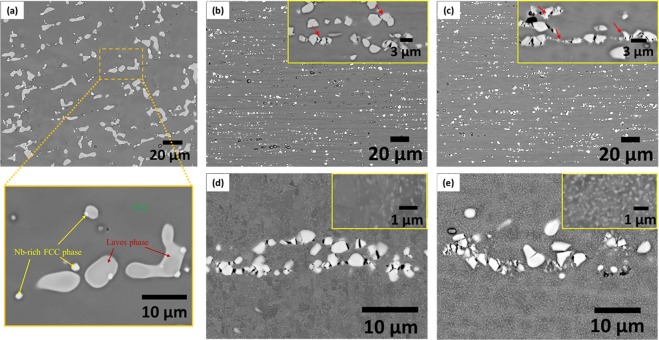
SEM micrographs of the CoCrFeNi_2.1_Nb_0.2_ HEA in the (**a**) homogenized (a magnified view shows the phases present); while (**b**) 90% cold-rolled, (**c**) 90% cryo-rolled, (**d**) 90% cold-rolled and annealed (800 °C/1 h) and (**e**) 90% cryo-rolled and annealed (800 °C/1 h) conditions (inset at the top right in the respective figures shows a magnified view).

The microstructure of the homogenized HEA (Fig. 1(a)) shows the presence of grey primary dendrites identified as the matrix FCC phase, while the bright phase appearing in-between the dendritic arm is the minor Laves phase (average size ~5–10 µm). The magnified view of the region marked in Fig. 1(a) further reveals the presence of another minor Nb-rich phase (Table 1), distinguished from the matrix and the minor Laves phase by their characteristic contrast differences. Interestingly, the minor Nb-rich phase appears mostly conjoined with the minor Laves phase. The microstructure of the 90% cold-rolled HEA (Fig. 1(b)) shows a preferred alignment of the fragmented Laves phase along the rolling direction (RD) (inset in Fig. 1(b) shows a magnified view) having average size reduced to ~1.00 ± 0.44 µm. The microstructure of the cryo-rolled HEA (Fig. 1(c)) appears rather similar to the cold-rolled HEA. However, the Laves phase particles show more severe fragmentation (marked by red arrows in the inset in Fig. 1(c)), resulting in a lower average size ~ 0.68 ± 0.40 µm. Upon annealing, the microstructures of both the annealed materials (Fig. 1(d,e)) show the presence of profuse nano-precipitates. The inset in the two micrographs qualitatively yet clearly indicates a higher density of precipitates in the cryo-rolled and annealed HEA (Fig. 1(e)).

**Table 1:** Chemical compositions (in at. %) of the different phases in the CoCrFeNi_2.1_Nb_0.2_ HEA after different thermo-mechanical treatments.

Condition	Phase	V_f_	Co	Cr	Fe	Ni	Nb
Nominal (Experimental)			18.87 (18.77 ± 0.30)	18.87 (20.17 ± 0.90)	18.87 (19.14 ± 0.51)	39.62 (38.41 ± 0.70)	3.77 (3.52 ± 0.39)
Starting material (homogenized at 1200 °C/24 h)	FCC	~92%	18.85 ± 0.05	21.11 ± 0.16	20.23 ± 0.19	38.26 ± 0.14	1.52 ± 0.42
	Laves (HCP)	~7%	17.13 ± 0.19	19.55 ± 0.21	15.20 ± 0.37	30.28 ± 0.43	17.85 ± 1.07
	Nb-rich FCC	~1%	15.66 ± 0.23	17.86 ± 0.40	10.26 ± 0.56	23.87 ± 0.66	34.01 ± 1.75
90% Cold-rolled and annealing at 800 °C/1 h	FCC	~82%	18.72 ± 0.07	20.61 ± 0.98	19.99 ± 0.66	38.88 ± 1.14	1.80 ± 0.55
	ε-phase (D0_19_)	~11%	18.55 ± 0.26	19.53 ± 1.48	18.90 ± 0.61	38.45 ± 2.43	4.58 ± 0.62
	Laves (HCP)	~6%	16.88 ± 0.45	19.84 ± 0.39	14.54 ± 0.76	29.32 ± 1.33	19.44 ± 2.86
	Nb-rich FCC	~1%	16.14 ± 0.53	16.73 ± 1.09	10.29 ± 1.08	25.94 ± 1.19	30.89 ± 3.09
90% Cryo-rolled and annealed at 800 °C/1 h	FCC	~75%	19.03 ± 0.40	20.90 ± 0.96	20.30 ± 0.30	38.20 ± 0.95	1.53 ± 0.23
	ε-phase (D0_19_)	~18%	18.62 ± 0.13	19.88 ± 0.59	18.72 ± 0.19	37.96 ± 2.18	4.82 ± 1.75
	Laves (HCP)	~6%	17.15 ± 0.38	18.90 ± 0.62	15.48 ± 1.04	29.83 ± 1.87	18.60 ± 3.85
	Nb-rich FCC	~1%	15.78 ± 0.40	16.76 ± 0.81	10.96 ± 1.72	23.72 ± 1.53	32.76 ± 4.26

**Figure 2:**
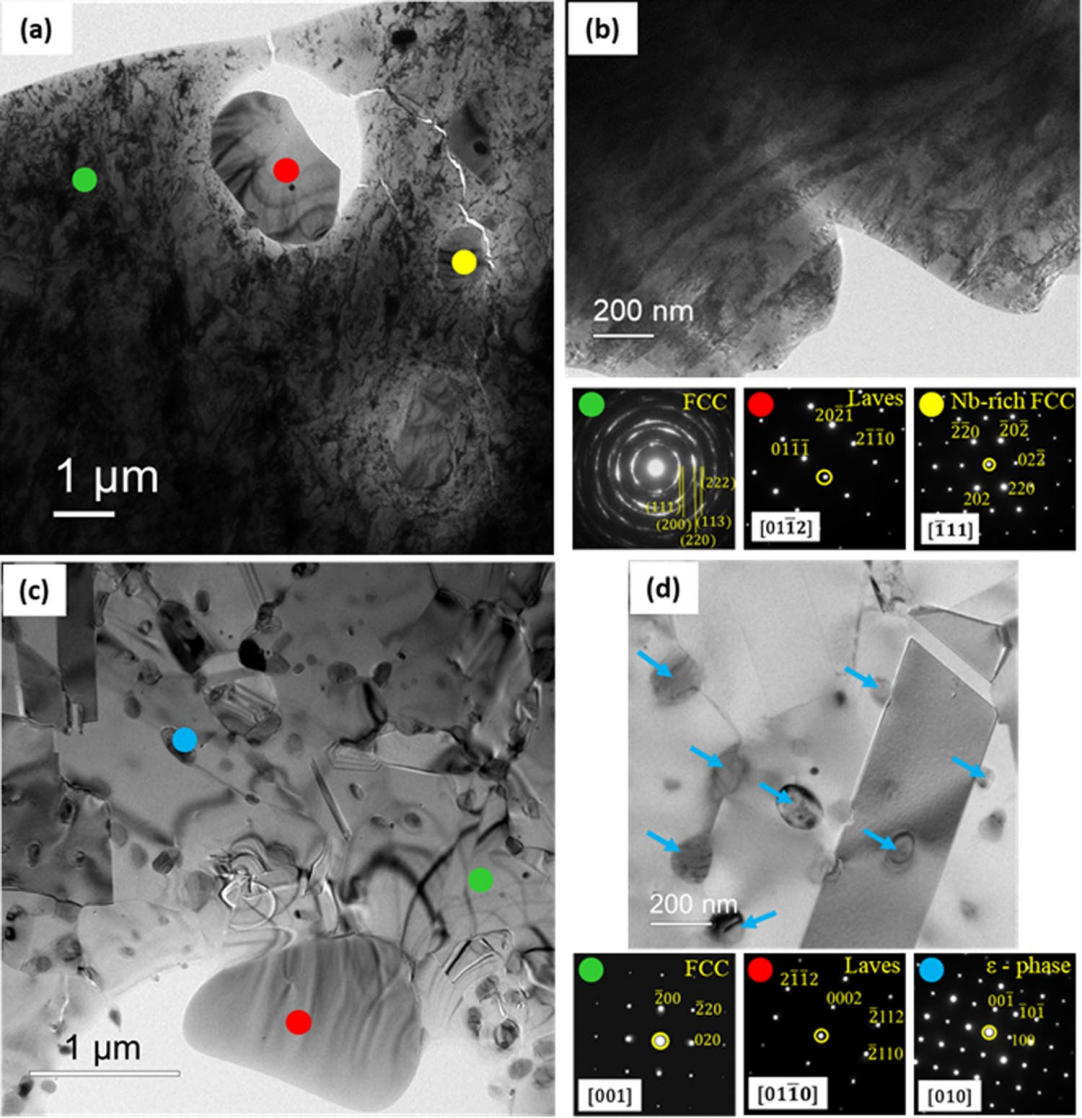
(**a**) TEM micrograph (observed on the RD-TD (Rolling Direction-Transverse Direction) plane along the ND (Normal Direction)) of the cold-rolled HEA, and the associated SADPs show nanocrystalline FCC (green spot), Laves phase (red spot) and Nb-rich FCC (yellow spot) phases; (**b**) TEM micrograph illustrating the microstructure of the cold-rolled HEA observed on the RD-ND (Rolling Direction-Normal Direction) plane along the TD (Transverse Direction); (**c**,**d**) TEM micrographs showing microstructural evolution in 90% cold-rolled and annealed (800 °C/1 h) HEA. The TEM micrographs and associated SADPs show the recrystallized FCC grains (green), Laves phase (red), and ε nano-precipitates (blue). The nano-precipitates are marked by blue arrows in (**d**) for clarity.

The TEM micrograph (Fig. 2(a)) and the associated selected area diffraction patterns (SADPs) of the cold-rolled HEA show the presence of ultrafine to nanocrystalline FCC (as confirmed by the ring pattern obtained from the green spot), Laves phase (red spot) and Nb-rich FCC phase (yellow spot). The TEM micrograph in Fig. 2(b) shows that the matrix FCC phase is subdivided at the nanoscale by extended high angle boundaries (HABs) with average spacing ~65 ± 12 nm. The TEM micrograph (Fig. 2(c)) and the associated SADPs of the annealed HEA show the presence of the equiaxed recrystallized major FCC phase (green spot), Laves phase (red spot) and D0_19_ structured ε nano-precipitates (blue spot). A magnified TEM micrograph in Fig. 2(d) shows that the precipitates are distributed at the grain boundaries, triple points, as well as interiors of the grains and annealing twins (indicated by the blue arrows).

**Figure 3:**
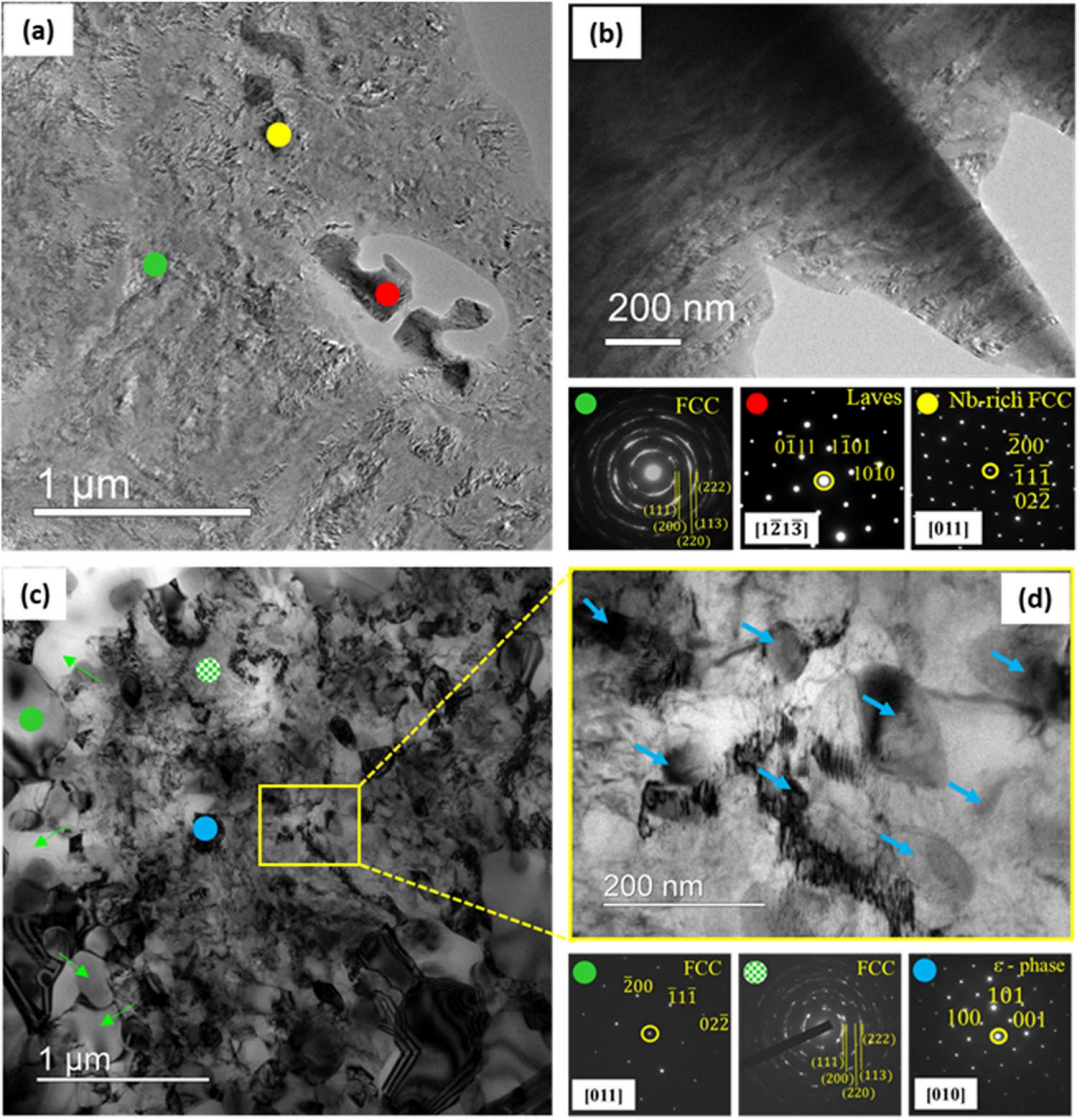
(**a**) TEM micrograph (observed on the RD-TD plane along the ND) of the cryo-rolled HEA and the associated SADPs show nanocrystalline FCC (green spot), Laves phase (red spot) and Nb-rich FCC (yellow spot) phases; (**b**) TEM micrograph illustrating the microstructure of the cryo-rolled HEA observed on the RD-ND plane along the TD; (c) TEM micrograph showing microstructure of the 90% cryo-rolled and annealed (800 °C/1 h) HEA. The TEM micrographs and associated SADPs show the recrystallized FCC grains (the SADP is obtained from the grain marked by the green circle, while green arrows identify the other recrystallized grains), retained deformed nanocrystalline FCC regions (hatched green circle), Laves phase (red), ε nano-precipitates (blue). (**d**) shows a magnified view of a region of interest in (marked by the square in (**c**)), which shows retained deformed regions dispersed with ε nano-precipitates (marked by blue arrows). The nano-precipitates are marked by blue arrows in (**d**) for clarity.

The TEM micrograph (Fig. 3(a)) and the associated SADPs of the cryo-rolled HEA confirm the presence of nanocrystalline FCC matrix (green spot), Laves phase (red spot) and Nb-rich phase (yellow spot). The TEM micrograph in Fig. 3(b) shows a lamellar structure of the FCC matrix subdivided at the nanoscale by HABs with average spacing ~50 ± 9 nm. The TEM micrograph of the annealed HEA (Fig. 3(c)) shows a remarkably heterogeneous microstructure featured by ultrafine recrystallized FCC grains (the associated SADP indicated by the green circle) as well as deformed nanocrystalline FCC matrix (the associated ring pattern indicated by the dotted green circle). ε nano-precipitates (the associated SADP indicated by the blue spot) are easily distinguished in the FCC matrix. A magnified view of a region of interest (marked by square) obtained from the deformed FCC region in Fig. 3(c) shows profuse ε nano-precipitates (indicated by blue arrows). Table 1 shows a higher fraction of the FCC matrix in the cold-rolled and annealed material (~82%) than that in the cryo-rolled and annealed material (~75%).

**Figure 4:**
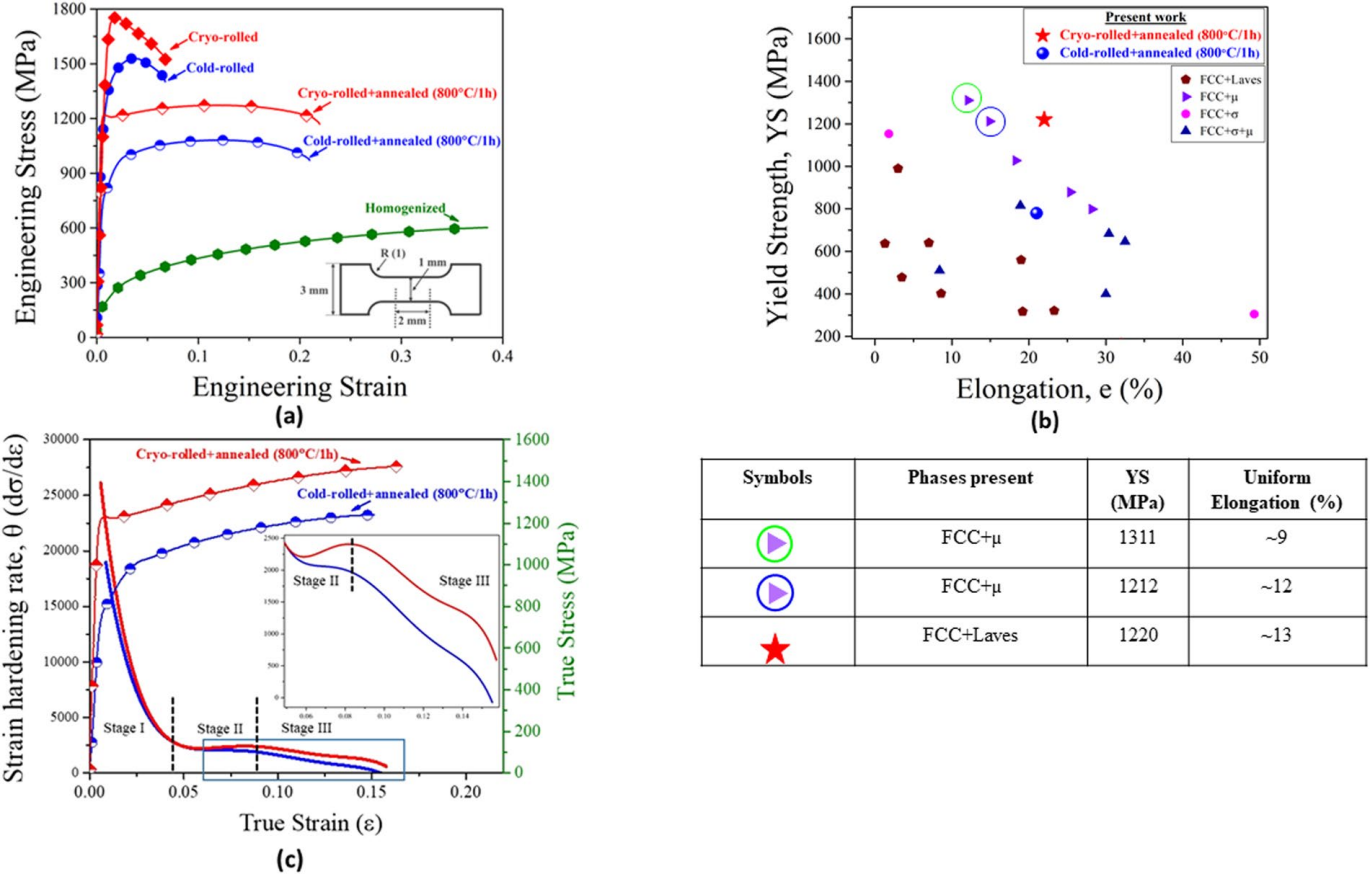
(**a**) Engineering stress-strain plots of CoCrFeNi_2.1_Nb_0.2_ HEA after different thermomechanical treatments (the dimensions of the tensile specimens are shown in the inset, and the tensile loading direction is along the RD); (**b**) The novel mechanical properties of the cryo-rolled and annealed HEA are compared with other selected brittle intermetallic containing HEAs in the YS vs. elongation plot (refer to the data in Supplementary). The uniform elongation associated with the data points enclosed by green and blue circles are further compared with that of the cryo-rolled+annealed HEA of the present research, as shown in the associated table placed below the figure (**b**); (**c**) shows the true stress and strain hardening rate as a function of true strain (inset shows the region of interest in (**c**) enclosed by the rectangle).

The mechanical properties of the HEA are compared in Fig. 4(a). The starting as-cast and homogenized HEA shows poor strength but large elongation. Both cryo- and cold-rolled HEAs show a huge increase in strength at the expense of elongation. Upon annealing, the cold-rolled and annealed HEA shows a good combination of strength (yield strength (YS): 774 ± 13 MPa; ultimate tensile strength (UTS): 1085 ± 18) and ductility (~20%). The remarkable enhancement in mechanical properties is obtained in the cryo-rolled and annealed HEA featured by an outstanding combination of strength (YS: 1219 ± 14 MPa, UTS: 1272 ± 25 MPa) and ductility (~22%). When compared to the cold-rolled and annealed HEA, the YS of the cryo-rolled and annealed HEA is increased by ~400 MPa (~58% increase), with an accompanying increase in elongation as well. Therefore, the cryo-rolled and annealed HEA shows a simultaneous improvement in strength and ductility when compared to the cold-rolled and annealed HEA. The novel mechanical properties of the cryo-rolled and annealed HEA are further compared with other brittle intermetallic containing HEAs in the YS vs. elongation plot in Fig. 4(b). In addition, the uniform elongation of selected high strength HEAs are compared in the associated table in Fig. 4(b). The cryo-rolled and annealed HEA is found to be amongst the HEAs with the best strength-ductility synergy.

To further understand the mechanical response, the true stress and work-hardening rate $(\theta  \sim \frac{d\sigma }{d\varepsilon })$ vs. true strain plots of both the annealed HEAs are shown in Fig. 4(c). Both the HEAs show a multistage work-hardening behavior. Following the usual drastic decrease in the work-hardening rate corresponding to the elastoplastic transition (Stage I), the work-hardening rate of the cold-rolled and annealed HEA decreases slowly (Stage II) before decreasing at an accelerated rate (Stage III). In stark contrast, the cryo-rolled and annealed material shows an increase in the work-hardening rate in Stage II, before starting to decrease in Stage III. Evidently, the cryo-rolled and annealed HEA with a heterogeneous microstructure maintains a significantly higher work-hardening rate throughout, as compared to the cold-rolled and annealed HEA with fully recrystallized microstructure.

**Figure 5:**
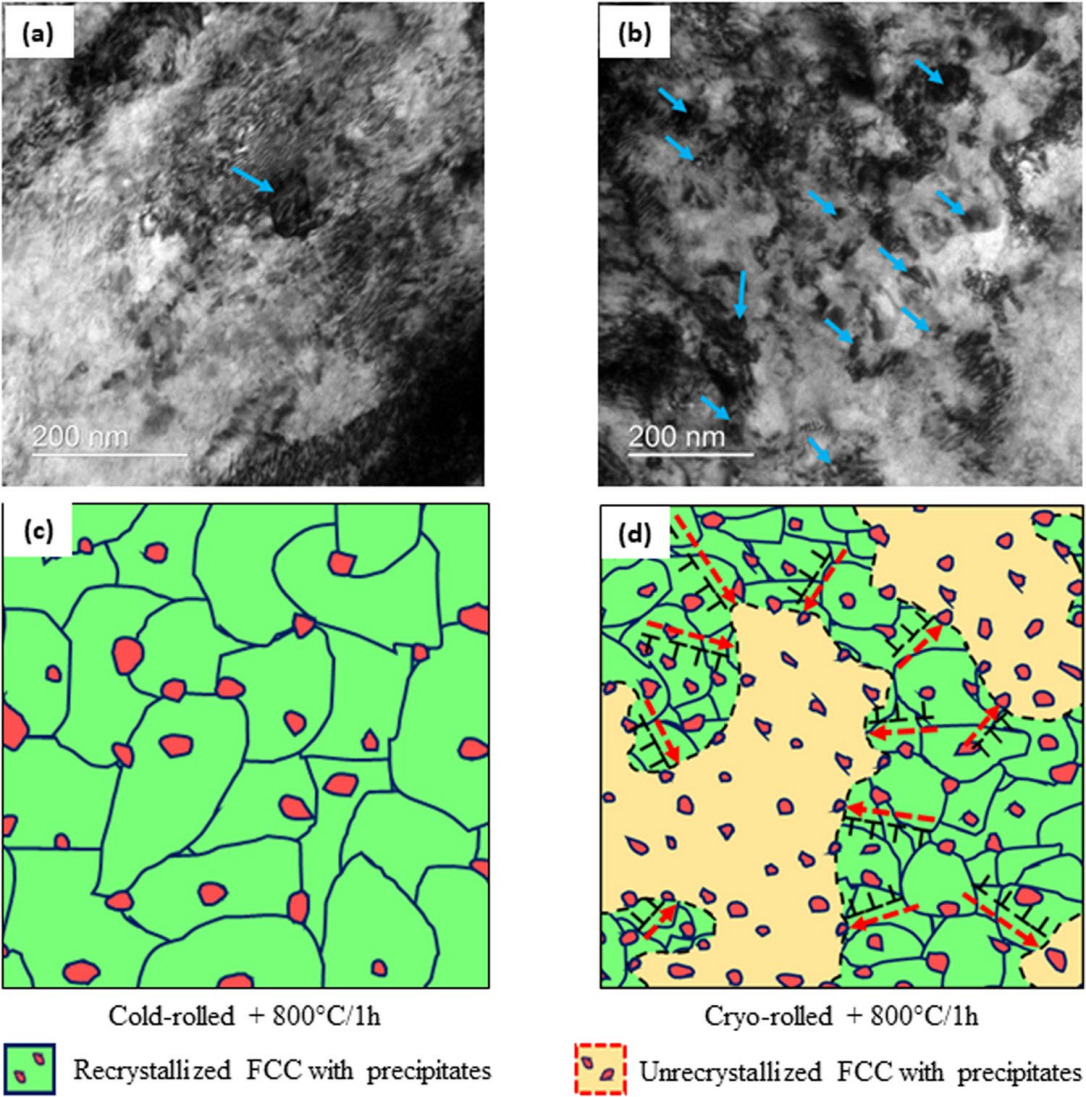
TEM microstructures of the (**a**) cold-rolled and (**b**) cryo-rolled HEA following annealing at 800 °C for 2 minutes. The precipitates are indicated by blue arrows. (**c**,**d**) show the schematic illustration of microstructural evolution upon recrystallization in the cold- and cryo-rolled HEA, respectively.

Therefore, the origin of the heterogeneous microstructure needs to be probed further. The evolution of the microstructure during annealing will be decided by the competing recrystallization and precipitation processes. Three different scenarios, namely precipitation occurring before recrystallization, concurrently with recrystallization, and post recrystallization, could be envisaged. To understand these aspects further, the microstructures of the cryo- and cold-rolled HEAs are compared after a short holding time of 120 seconds at 800 °C (Fig. 5(a,b)), which corresponds to an early stage of recrystallization. Even at this initial stage of recrystallization, the cryo-rolled HEA shows profuse ε nano-precipitates (marked by blue arrows in Fig. 5(b)), which are decidedly scarce in the cold-rolled HEA (Fig. 5(a)), indicating that precipitation kinetics clearly dominates over the recrystallization kinetics in the cryo-rolled HEA. The formation of the nano-precipitates can effectively pin the migration of the HABs, thereby significantly inhibiting the progress of recrystallization due to the Zener pinning effect. Notably, rather similar behavior is found in several age-hardening aluminum alloys in which heterogeneous precipitation inhibits the progress of recrystallization effectively^37–39^. In the cold-rolled materials, although the precipitates are found at the HABs and triple points, the microstructure is predominantly recrystallized, thus indicating that precipitation lags recrystallization. The contrasting precipitation behavior in the cold- and cryo-rolled material leads to the formation of the heterogeneous microstructure featured by co-existing deformed and recrystallized regions (Fig. 5(d)) in the cryo-rolled HEA, as opposed to a predominantly recrystallized microstructure in the cold-rolled HEA (Fig. 5(c)). It appears that the greater density of the potential heterogeneous nucleation sites (dislocations, shear bands, and grain boundaries) in the cryo-rolled HEA accelerates the precipitation process. The accelerated nucleation and overall precipitation kinetics in the cryo-rolled HEA leads to a higher volume fraction of precipitates, smaller precipitate diameter, and smaller interparticle spacing (Table 2) after the same isothermal holding time of 1 h at 800 °C.

**Table 2:** Differences in key parameters affecting the mechanical properties of the CoCrFeNi_2.1_Nb_0.2_ HEA.

Phase	Key parameters	Cold-rolled and annealed (800 °C/1 h)	Cryo-rolled and annealed (800 °C/1 h)
FCC	Grain size, nm	~780 ± 60	~450 ± 20
	Phase fraction	~82%	~75%
ε-phase	Diameter (d), nm	~125 ± 40	~97 ± 36
	Interparticle spacing (λ), nm	~420 ± 26	~200 ± 12
	Phase fraction	~11%	~18%

As already highlighted, microstructural tailoring, including ultrafine and nanostructuring, leads to remarkable enhancement in strength but usually at the loss of ductility due to the early onset of plastic instability. Therefore, the microstructural origin of the remarkable improvement in the strength-ductility balance in the cryo-rolled and annealed HEA needs to be understood. The strength of the HEAs can be expressed by:

1 $$\sigma ={\sigma }_{0}+{\sigma }_{D}+{\sigma }_{G}+{\sigma }_{P}$$

where, *σ*_0_ = lattice friction stress, *σ*_*D*_ = dislocation strengthening, *σ*_*G*_ = grain size strengthening and *σ*_*P*_ = precipitation strengthening. From Eq. 1, the difference in the strength (Δ*σ*) should be given by:

2 $$\Delta \sigma =\Delta \,{\sigma }_{D}+\Delta \,{\sigma }_{G}+\Delta \,{\sigma }_{P}$$

It is implied that much higher strength of the cryo-rolled and annealed HEA is contributed by the grain size, precipitation, and dislocation strengthening (from the retained hard deformed regions). However, simultaneous improvement in the strength-ductility synergy of the cryo-rolled and annealed originates from the novel heterogeneous microstructure. It is perceived that mechanical incompatibility between the different hardness domains in the heterogeneous microstructure enables the development of strain gradients at the vicinity of the interfaces/domain boundaries during deformation, which needs to be accommodated by the creation of geometrically necessary dislocations (GNDs)^14,40^. The pile-up of GNDs at the interfaces/domain boundaries, in turn, results in the development of long-range back stress, making the dislocations difficult to move in the softer recrystallized grains until the surrounding hard regions start yielding (Fig. 5(d)). Under such constrained conditions, the softer recrystallized grains appear much stronger than unconstrained deformation, leading to a considerable increase in the global yield strength^19^. The work-hardening associated with the back-stress opposes the early onset of necking, rendering improved strength-ductility synergy^14,16,41^. Although the back stress has not been quantified, the greater sustained work-hardening rate of the cryo-rolled and annealed HEA as compared to that in the cold-rolled and annealed HEA (Fig. 4(c)), even at a much higher level of strength, is indicative of the critical role of the heterogeneous microstructure.

It is interesting to note that monotonic cryo-rolling^42^ and novel variants such as hybrid-rolling (cryo- followed by warm-rolling)^43^ have been exploited by us in our earlier research to achieve novel structural and/or compositionally heterogeneous microstructure in multiphase lamellar eutectic AlCoCrFeNi_2.1_ HEA. The vastly different hardness of the constituent phases results in differential strain-partitioning, which in turn, results in differential stored energy or driving force for recrystallization during annealing and warm-rolling. In contrast, in the present research, the same cryo-rolling is exploited to tune the heterogeneous precipitation behavior for achieving the heterogeneous microstructure. These results clearly indicate significant opportunities for achieving novel heterogeneous microstructures in a wide range of HEAs through innovative processing by cryo-rolling.

In summary, homogenized CoCrFeNi_2.1_Nb_0.2_ HEA containing complex Laves phase was severely cold- or cryo-rolled and annealed. Cryo-rolling resulted in a finer lamellar nanostructure and greater fragmentation of the Laves phase. Annealing resulted in the precipitation of the ε phase. However, the cold-rolled and annealed HEA showed a predominantly recrystallized microstructure, in stark contrast to a heterogeneous microstructure featured by deformed and ultrafine recrystallized FCC grains in the cryo-rolled and annealed HEA. The heterogeneous microstructure appeared to originate from the accelerated precipitation of the nanosized ε phase early in the recrystallization process, thus considerably inhibiting the progress of recrystallization. The heterogeneous microstructure of cryo-rolled and annealed HEA resulted in excellent strength-ductility synergy. The present study could open processing and microstructural design pathways for attaining novel mechanical properties even in brittle intermetallic containing HEAs.

## Methods

Button-shaped melts (~50 mm × 8 mm) of the CoCrFeNi_2.1_Nb_0.2_ HEA were prepared by arc-melting under inert (argon) atmosphere starting with high purity (≥99.9%) elements. The ingots were flipped and re-melted at least for five times to achieve greater chemical homogeneity. Samples (25 mm × 8 mm × 4 mm) were extracted from the homogenized (1200 °C/24 h) buttons and were multi-pass cold- and cryo-rolled up to 90% reduction in thickness (final thickness ~0.4 mm) using a laboratory-scale rolling mill (SPX Precision Instruments, USA). During cryo-rolling, the specimens were immersed in liquid N_2_ for 0.5 h before and after every pass. The cold- and cryo-rolled sheets were subsequently annealed at 800 °C for 1 h, followed by water quenching.

The microstructural analyses were accomplished using FEG-SEM (Model: JSM-7800F; Make: JEOL, Japan) equipped with energy-dispersive x-ray spectroscopy (EDS) (EDAX Inc., USA) and electron backscattering diffraction (EBSD) (EDAX Inc., USA) attachments. Further phase and microstructural analyses were carried out by JEM-2100 (JEOL, Japan) and Tecnai G2 (FEI, Netherlands) TEMs; both operated at 200 kV. Chemical compositions of the different phase constituents were determined using energy dispersive spectroscopy (EDS) analysis (EDAX Inc., USA). The specimens for TEM analyses were prepared by standard mechanical polishing followed by electropolishing (electrolyte: 10% perchloric acid in ethanol; temperature: −10 °C; voltage: 20 V). The cross-sectional TEM specimens were prepared by the Ar+ ion milling process using the precision ion polishing system (PIPS) (Model: 691; Make: Gatan Inc.) with a beam acceleration voltage of 5 kV and the incident angle of ±4°. Selected area electron diffraction (SAED) patterns were indexed using the CrysTBox diffractGUI software package^44^. The volume fractions of different constituent phases were determined from several SEM backscattered electron images using ImageJ analysis software^45^.

The dog-bone shaped specimens prepared using wire electrical discharge machining (EDM) were used for the tensile tests. The surfaces of the tensile specimens were carefully mechanically polished to minimize surface inhomogeneities. Tensile tests were conducted using a standard table-top universal testing machine (UTM) (Model: INSTRON 5967; Make: INSTRON Inc., USA) at ambient temperature using an initial strain rate of 1 × 10^−3^ s^−1^. The commercial VIC-2D high-speed Digital Image Correlation (DIC) system (Correlated Solutions Inc., USA) was used for precisely measuring the engineering strain by means of creating the very fine speckled pattern of black dots randomly distributed over the white backgrounds. The images were acquired using a commercial Vic-Snap 9 acquisition system and analyzed using a subset size of 31 and a step size of 7 for fine resolution. The engineering stress-strain curves were plotted from the obtained stress (load cell) and strain (DIC analysis) data.

## Supplementary information

Supplementary Data.

## Data Availability

The dataset generated during and/or analyzed during the current study are not publicly available as they form part of an ongoing study but are available from the corresponding author on reasonable request.
