# Supplementary material for: Heterogeneous precipitation mediated heterogeneous nanostructure enhances strength-ductility synergy in severely cryo-rolled and annealed CoCrFeNi2.1Nb0.2 high entropy alloy
Source: Sci Rep. 2020 Apr 8;10:6056. doi: 10.1038/s41598-020-63038-z (PMC7142141; doi:10.1038/s41598-020-63038-z)
Supplement: Supplementary file 1 — Supplementary Data. [file 41598_2020_63038_MOESM1_ESM.docx]

**Supplementary data**

**Heterogeneous precipitation mediated heterogeneous nanostructure enhances strength-ductility synergy in severely cryo-rolled and annealed CoCrFeNi_2.1_Nb_0.2_high entropy alloy**

U. Sunkari^1^, S. R. Reddy^1^, B. D. S. Rathod^1^, S.S. Satheesh Kumar^2^, R. Saha^3^, S. Chatterjee^1^, P. P. Bhattacharjee^1,*^

^1^Indian Institute of Technology Hyderabad, Kandi, Sangareddy 502285, Telangana, India

^2^Defence Metallurgical Research Laboratory, Hyderabad 500058, Telangana, India

^3^R&D Division, TATA Steel, Jamshedpur 831001, India

Table S1: Properties of selected HEAs containing intermetallic phases (data used in plotting Fig.4(b))

| **S.No.** | **System** | **Alloy Designation** | **Condition** | **YS** | **UTS** | **%El** | **Phases** | **Ref.** |
| --- | --- | --- | --- | --- | --- | --- | --- | --- |
| 1 | CoCrFeNiNb_x_ | CoCrFeNiNb_0.103_ | as-cast | 317 | 622 | 19.2 | FCC+Laves | [1] |
|  |  | CoCrFeNiNb_0.155_ | as-cast | 321 | 744 | 23.3 | FCC+Laves |  |
|  |  | CoCrFeNiNb_0.206_ | as-cast | 402 | 807 | 8.6 | FCC+Laves |  |
|  |  | CoCrFeNiNb_0.309_ | as-cast | 478 | 879 | 3.5 | FCC+Laves |  |
|  |  | CoCrFeNiNb_0.412_ | as-cast | 637 | 1004 | 1.3 | FCC+Laves |  |
| 2 | CoCrFeNi_2.1_Nb_x_ | CoCrFeNi_2.1_Nb_0.2_ | as-cast | 170 | 537 | 32 | FCC+Laves | [2] |
|  |  |  | as-cast+800°C/24h | 560 | 860 | 19 | FCC+Laves+ε |  |
|  |  | CoCrFeNi_2.1_Nb_0.4_ | as-cast | 640 | 840 | 7 | FCC+Laves |  |
|  |  |  | as-cast+800°C/24h | 990 | 1160 | 3 | FCC+Laves+ε |  |
| 3 | Cr_15_Fe_20_Co_35_Ni_20_Mo_10_ (Mo_10_) | Cr_0.75_FeCo_1.75_NiMo_0.5_ | 1100°C HR and CR (70%)+800 °C/1 h/Air Cooled | 1311 | 1410 | 12.1 | FCC+µ | [3] |
|  |  |  | 1100°C HR and CR (70%)+850 °C/5 min/Water Quenched | 1212 | 1360 | 14.9 | FCC+µ |  |
|  |  |  | 1100°C HR and CR (70%)+900 °C/5 min/Water Quenched | 1028 | 1249 | 18.3 | FCC+µ |  |
|  |  |  | 1100°C HR and CR (70%)+1000 °C/5 min/Water Quenched | 879 | 1194 | 25.4 | FCC+µ |  |
|  |  |  | 1100°C HR and CR (70%)+1000 °C/1 h/Air Cooled | 799 | 1127 | 28.2 | FCC+µ |  |
|  |  |  | 1100°C HR and CR (70%)+1150 °C/1 h/Air Cooled | 350 | 918 | 62.4 | FCC+µ |  |
| 4 | CoCrFeMoNi | CoCrFeMo_0.3_Ni | as-cast+500°C/4h | 305.3 | 709.7 | 49.3 | FCC+σ | [4] |
|  |  |  | as-cast+CR(60%)+850°C/1h | 815.5 | 1186.5 | 18.9 | FCC+σ +µ |  |
|  |  |  | as-cast+CR(60%)+950°C/5h | 646.7 | 1042.0 | 32.5 | FCC+σ +µ |  |
|  |  |  | as-cast+CR(60%)+950°C/5h++700°C/5h | 683.7 | 1066.6 | 30.4 | FCC+σ+µ |  |
| 5 | CoCrFeNiV | CoCrFeNiV | as-cast | -- | 311 | 0 | FCC+σ | [5] |
|  |  | CoCrFeNiV | as-cast+1000°C/24h | -- | 330 | 0 | FCC+σ |  |
| 6 | CoCrFeNi_2.1_Nb_x_ | CoCrFeNi_2.1_Nb_0.2_ | as-cast+1200°C/24h+CR(90%) | 1380 | 1530 | 7 | FCC+Laves | Present work |
|  |  |  | as-cast+1200°C/24h+CR(90%)+800°C/1h | 780 | 1080 | 21 | FCC+Laves+ε |  |
|  |  |  | as-cast+1200°C/24h+CY(90%) | 1680 | 1760 | 10.94 | FCC+Laves |  |
|  |  |  | as-cast+1200°C/24h+CY(90%)+800°C/1h | 1220 | 1270 | 22 | FCC+Laves+ε |  |
| 7 | Ni_14_Fe_20_Cr_26_Co_20_Mn_20_ | Ni_0.7_FeCr_1.3_Co_1_Mn | as-cast + 1000°C/24h + CR(80%)+675°C/1h | 1153 | 1187 | 1.8 | FCC+σ | [6] |
| 8 | CoCrFeNiMo_x_ | CoCrFeNiMo_0.5_ | as-cast + 900°C/1h | 510.5 | 731.7 | 8.4 | FCC+σ+µ | [7] |
| 9 | CoCrFeNiMo_0.2_ | CoCrFeNiMo_0.2_ | as-extruded+ 800°C/72h | 400 | 850 | 30 | FCC+σ+µ | [8] |
| 10 | Al_5_Nb_24_Ti_40_V_5_Zr_26_ alloy | Al_5_Nb_24_Ti_40_V_5_Zr_26_ alloy | as-cast +CR(80%)+ annealing at 800 °C/1h | 835 | 880 | 26.7 | BCC+Laves | [9] |
| 11 | CoCrFeNiNb_x_ | CoCrFeNiNb_0.1_ | as-deposited (direct laser deposition) | 380 | 650 | 52 | FCC+Laves | [10] |
|  |  | CoCrFeNiNb_0.15_ | as-deposited (direct laser deposition) | 580 | 710 | 36 | FCC+Laves |  |
|  |  | CoCrFeNiNb_0.2_ | as-deposited (direct laser deposition) | 730 | 840 | 10 | FCC+Laves |  |

**References**

[1] W.H. Liu, J.Y. He, H.L. Huang, H. Wang, Z.P. Lu, C.T. Liu, Effects of Nb additions on the microstructure and mechanical property of CoCrFeNi high-entropy alloys, Intermetallics 60 (2015) 1-8.

[2] U. Sunkari, S.R. Reddy, S. Chatterjee, P.P. Bhattacharjee, Effect of prolonged aging on phase evolution and mechanical properties of intermetallic strengthened CoCrFeNi2.1Nbx high entropy alloys, Materials Letters 248 (2019) 119-122.

[3] K. Ming, X. Bi, J. Wang, Precipitation strengthening of ductile Cr15Fe20Co35Ni20Mo10 alloys, Scripta Materialia 137 (2017) 88-93.

[4] W.H. Liu, Z.P. Lu, J.Y. He, J.H. Luan, Z.J. Wang, B. Liu, Y. Liu, M.W. Chen, C.T. Liu, Ductile CoCrFeNiMox high entropy alloys strengthened by hard intermetallic phases, Acta Materialia 116 (2016) 332-342.

[5] G.A. Salishchev, M.A. Tikhonovsky, D.G. Shaysultanov, N.D. Stepanov, A.V. Kuznetsov, I.V. Kolodiy, A.S. Tortika, O.N. Senkov, Effect of Mn and V on structure and mechanical properties of high-entropy alloys based on CoCrFeNi system, Journal of Alloys and Compounds 591 (2014) 11-21.

[6] A.J. Zaddach, R.O. Scattergood, C.C. Koch, Tensile properties of low-stacking fault energy high-entropy alloys, Materials Science and Engineering: A 636 (2015) 373-378.

[7] Z. Niu, Y. Wang, C. Geng, J. Xu, Y. Wang, Microstructural evolution, mechanical and corrosion behaviors of as-annealed CoCrFeNiMox (x = 0, 0.2, 0.5, 0.8, 1) high entropy alloys, Journal of Alloys and Compounds 820 (2020) 153273.

[8] C. Zhang, B. Liu, Y. Liu, Q. Fang, W. Guo, H. Yang, Effects of Annealing on Microstructure and Mechanical Properties of Metastable Powder Metallurgy CoCrFeNiMo0. 2 High Entropy Alloy, Entropy 21(5) (2019) 448.

[9] S. Zherebtsov, N. Yurchenko, E. Panina, M. Tikhonovsky, N. Stepanov, Gum-like mechanical behavior of a partially ordered Al5Nb24Ti40V5Zr26 high entropy alloy, Intermetallics 116 (2020) 106652.

[10] K. Zhou, J. Li, L. Wang, H. Yang, Z. Wang, J. Wang, Direct laser deposited bulk CoCrFeNiNbx high entropy alloys, Intermetallics 114 (2019) 106592.
